# Use of self-organizing maps for analyzing the behavior of canines displaced towards midline under interceptive treatment

**DOI:** 10.4317/medoral.21509

**Published:** 2017-02-04

**Authors:** Vicente Gandía-Aguiló, Rosa Cibrián, Emilio Soria, Antonio-José Serrano, Luz Aguiló, Vanessa Paredes, Jose-Luis Gandía

**Affiliations:** 1 Orthodontics. Department of Stomatology, Faculty of Medicine and Dentistry, University of Valencia, Valencia, Spain; 2 Medical Physic Unit. Department of Physiology University of Valencia, Valencia, Spain; 3 IDAL (Intelligent Data Analysis Laboratory). Department of Electronic Engeenering. ESTE. University of Valencia, Valencia, Spain; 4 Private office (Doctores Gandía & Aguiló - Identis) in Valencia

## Abstract

**Background:**

Displaced maxillary permanent canine is one of the more frequent findings in canine eruption process and it’s easy to be outlined and early diagnosed by means of x-ray images. Late diagnosis frequently needs surgery to rescue the impacted permanent canine. In many cases, interceptive treatment to redirect canine eruption is needed. However, some patients treated by interceptive means end up requiring fenestration to orthodontically guide the canine to its normal occlusal position. It would be interesting, therefore, to discover the dental characteristics of patients who will need additional surgical treatment to interceptive treatment.

**Material and Methods:**

To study the dental characteristics associated with canine impaction, conventional statistics have traditionally been used. This approach, although serving to illustrate many features of this problem, has not provided a satisfactory response or not provided an overall idea of the characteristics of these types of patients, each one of them with their own particular set of variables. Faced with this situation, and in order to analyze the problem of impaction despite interceptive treatment, we have used an alternative method for representing the variables that have an influence on this syndrome. This method is known as Self-Organizing Maps (SOM), a method used for analyzing problems with multiple variables.

**Results:**

We analyzed 78 patients with a PMC angulation higher than 100º. All of them were subject to interceptive treatment and in 21 cases it was necessary to undertake the above-mentioned fenestration to achieve the final eruption of the canine.

**Conclusions:**

In this study, we describe the process of debugging variables and selecting the appropriate number of cells in SOM so as to adequately visualize the problem posed and the dental characteristics of patients with regard to a greater or lesser probability of the need for fenestration.

**Key words:**Interceptive orthodontic treatment, altered eruption, impacted canines, neuronal networks, self-organizing maps.

## Introduction

The permanent maxillary canine (PMC) is the tooth that presents the highest incidence of displacement from its normal eruptive path due to its peculiar morphological, topographical and chronological characteristics. Its inclination varies with age, nine years old being when it presents maximum inclination, 99º (1). Theories related with the etiology of impacted canines and predictive variables of canine impaction in the mixed dentition are reviewed with an insight into current interceptive treatment modalities (2). Diagnosing canines with eruptive alterations is undertaken clinically and radiographically using panoramic radiographs (3).

Interceptive treatment is focused on improving the altered eruptive path so as to achieve the eruption of the canine (4-6), but some patients end up also requiring surgical help.

The decision to undertake a fenestration on a patient subjected to interceptive treatment is a decision taken by a specialist based on clinical experience. Hence, in this work, our aim was to find the pattern or patterns of those patients with displaced canines towards the midline for whom interceptive orthodontic treatment is not sufficient for the correct eruption of the canine.

Given the great number of variables that can affect this (7-13), it is not possible using traditional methods to visualize the distribution of these variables in the sample studied and to determine patterns of behavior that may explain which cases have a greater or lesser probability of the need for fenestration in order to achieve the eruption of the canine. In this study we have used an alternative methodology - self-organizing maps (SOM), whose application to this problem will be detailed in the following section on material and methods. This method will allow us to group the study patients into a certain number of cells, called neurons. The algorithm of the SOM will determine the value of each study variable corresponding to each of those neurons. By doing so, it is possible to simultaneously visualize the value of each variable involved in the study, in each group of patients included in a neuron. Using this grouping method, we can visualize all the variables in the distinct groups of patients and find behavior patterns associated with a certain variable, in our case the need for undertaking a fenestration.

## Material and Methods

The design of this research article was approved by the University of Valencia Ethics Committee on Human Research (H1462653247069). Rights have been protected by the relevant Institutional Review Board and written informed consent to participate was granted by all subjects. The study followed guidelines established by the Helsinki Declaration for research involving human subjects, as well as STROBE guidelines.

78 panoramic radiographs were analyzed of patients with a pathology associated with the inclusion of permanent canines, of ages between 6 and 13 years at the beginning of treatment, of which 50 were girls and 28 boys.

A panoramic radiograph was available for all cases at the beginning of treatment and another of the final solution in which it was shown that the canine had erupted, regardless of whether or not there was a need for fenestration.

**Figure 1:**
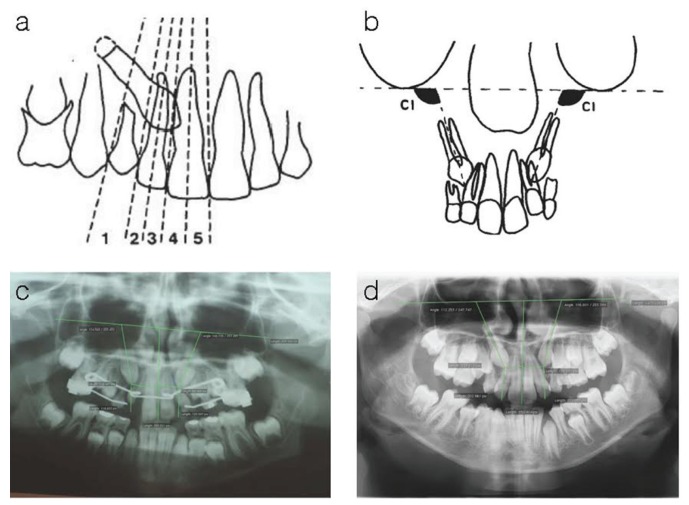
Localization and determination of the PMC. (a) Sector of canine location (b, c) Determining the angles of the canines (d) Fanned incisors.

The selection criterion was that the patients had to have a PMC angulation higher than the norm, i.e. greater than 100º (1). This measurement is reflected in the box marked INC 13 INC 23 in  Table 1 and figure. 1b,c.

**Table 1:**
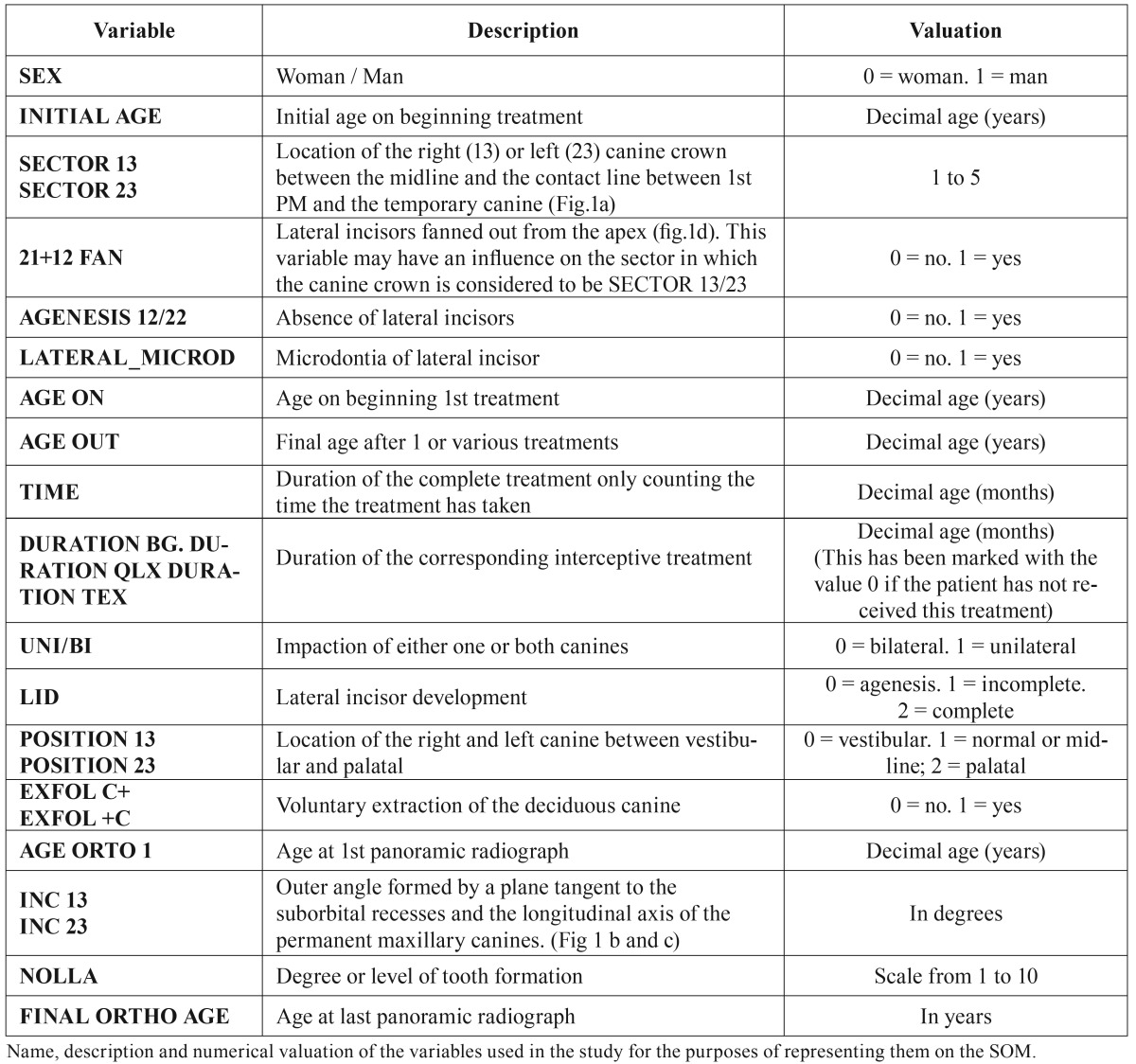
Variables represented on the SOM.

The 3 types of interceptive appliances used were: Gosgharian transpalatal bar type (BG); Quad Helix (QLX); Extraoral Traction (TEX).

The study variables were obtained from the initial and final panoramic radiographs of each patient and from their clinical records. The OsiriX software was used for taking measurements on the panoramic radiograph. While numerical variables were considered with their measured value, non-numerical or categorical variables were assigned with a numerical value in order to allow them to be represented on the SOM.  Table 1 presents the variables considered in the study along with their assigned values.

- Self Organizing Maps

Self Organizing Maps (SOM) (14) are one of the most popular visualization tool nowadays. SOM is an Artificial Neural Network (ANN) proposed by Teuvo Kohonen (15) and, since then, it has been analyzed and employed extensively in a wide variety of domains, such medical (16,17), engineering applications (18,19) and even in the field of animal sciences (20).

In contrast to SOMs, classical techniques can only deal with accurate visualizations of whole data sets when the number of features required is equal or lower than three; for a higher number of features to be represented, only projections onto three dimensions can be carried out, establishing restrictions (such as keeping fixed certain sets of variables and representing the rest). Such a restriction leads to a partial representation of the information. Moreover, most of the real data sets are formed by more than three features, making graphical representation difficult. For the type of representation that will make it possible to find and visualize patterns in multiple dimension data sets, self-organizing maps (SOM) are especially recommendable.

To obtain a SOM, each patient is represented by a vector of as many coordinates and variables that are to be considered, in our case 25 variables initially ( Table 1). By means of an iterative algorithm, the patients are grouped into blocks called neurons, the purpose of which is that the patients who make up each of these neurons have similar characteristics and different ones from those making up other neurons.

The number of neurons and, therefore, of groups to be formed is a choice that must be taken at the beginning of the SOM. The number of neurons may vary from a few dozen up to several thousands. In our case we chose a set of 25 neurons, appropriate for the number of patients and variables to be studied.

**Figure 2:**
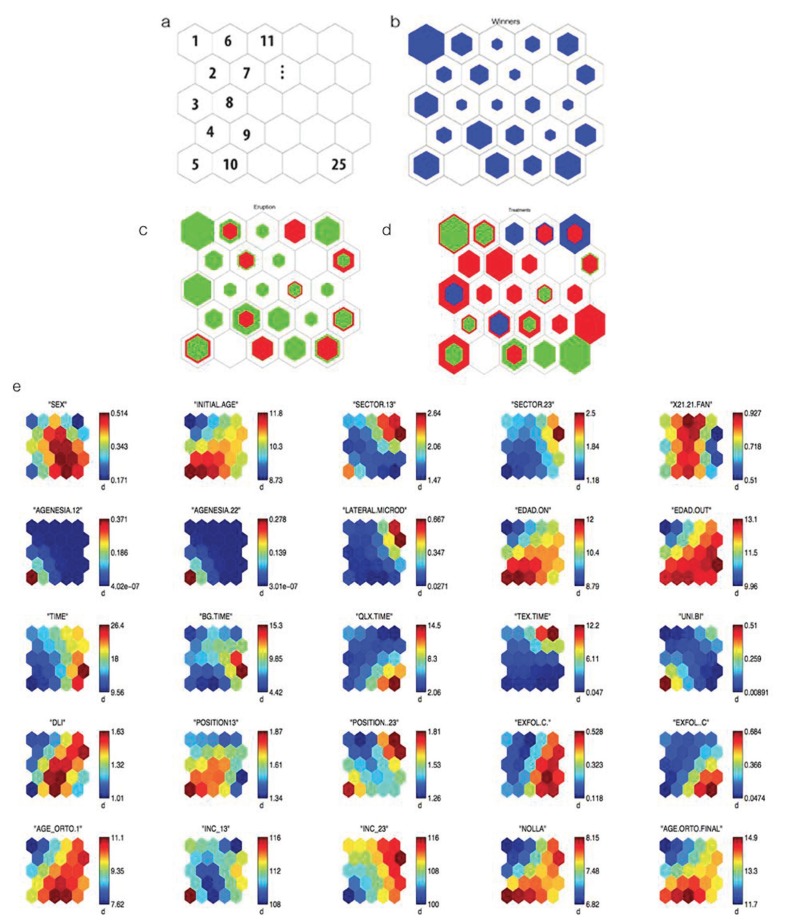
Distribution of SOM. a) Numbering of the neurons: Conventionally, neurons are numbered from top to bottom and left to right. b) Distribution of the study patients into each of the neurons, taking the 25 variables of the study into account. Empty neurons correspond to patterns that do not fit any patient and greater or lesser amount of blue indicates the greater or lesser number of patients in each neuron. c) Distribution of cases that required fenestration (in red) and those that only needed interceptive treatment (in green) in each of the neurons. The empty neurons correspond to patterns that were non-existent in our study. d) Distribution of treatments (BG in red QLX in green and TEX blue) in each of the neurons. e) Distribution of the values of the study variables in each of the neurons that contain a pattern of patients with a similar minimum distance according to the Artificial Neural Network algorithm. The description and valuation of the variables represented are shown in Table 1.

After undergoing the SOM training process, the patients are distributed on the map, which shows that some neurons have a greater number of patients than others or that there are even empty neurons (Fig. 2a,b). Empty neurons show that none of our patients have the pattern corresponding to that neuron.

It is now possible to project the map onto the different features; these projections are called component planes, also known as component maps (Fig. 2e). A component plane of a SOM is a map where, for each neuron, only one component of the vector representing the pattern corresponding to that neuron is displayed (Fig. 2e for instance). So, for 25 variables, 25 component maps are obtained and in each of them one can visualize how the value of the variable changes in the different neurons that make up the maps and, therefore, in the different patterns of patients that have been determined.

Each component plane is shown next to a colored bar which gives information about the relationship between the color and the corresponding numerical value.

## Results

As mentioned in the previous section, each patient is represented by a vector of as many components and variables as there are in the study: initially 25 variables. An analysis of the minimum distances between those vectors allows us to group the patients into neurons when they have similar characteristics. In our study, due to the number of cases involved, a map of 25 neurons was used in which the 78 patients were distributed into the neurons as shown in figure. 2a,b.

Using the criteria indicated in the material and methods section, where a greater percentage of filling in of the neuron indicates a greater number of patients with that pattern and an empty neuron indicates that there are no cases with the characteristics that correspond to that neuron, we can observe that neuron 1 with 12 patients, and 5, 9 and 25 with 6 patients each are the neurons with the greatest number of patients. The other neurons contain between 1 and 5 patients and neurons 10 and 17 are empty. Therefore, each neuron shows the different patients patterns that can be found in our study.

This same scheme (Fig. 2c) shows how the patients on whom it was necessary to undertake a fenestration in order to achieve the eruption of the canine (in red) are distributed in each neuron or if the canine has erupted with interceptive orthodontic treatment (in green).

Hence, it can be observed that in some neurons all or practically all the canines of the patients have erupted, whereas, in other neurons, the contrary is true and in a third group it can be observed that there is practically the same number of cases that required fenestration as cases that had eruptions following orthodontic treatment.

Figure 2d shows the distribution of the orthodontic treatments employed (BG, QHX and TEX) in each neuron.

Lastly, (Fig. 2e) shows, for each of the 25 variables included in the study, the distribution on a color scale of the value of each of these magnitudes in the pattern corresponding to each neuron. In this case it can be observed that all the neurons, including the empty ones have an assigned value for the variable indicated because the method has extrapolated the value of the variable that would correspond to that neuron. Given that in our study there were no patients assigned to those neurons, the pattern associated with those empty neurons is of no interest to our study.

**Figure 3:**
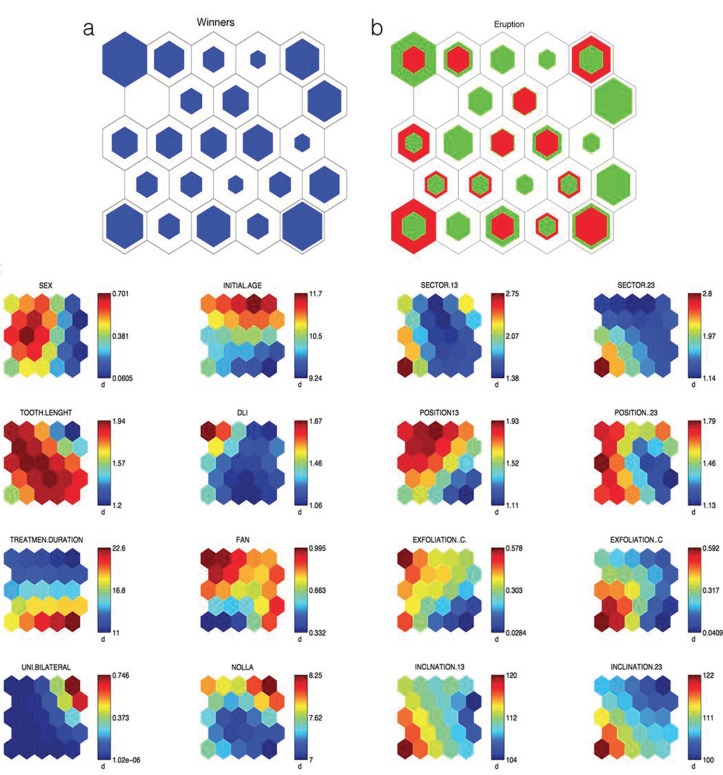
New Distribution of SOM. (a) Distribution of study patients (b) Distribution of the cases that required fenestration (in red) and those that only needed interceptive treatment (in green). Empty neurons correspond to patterns where there is no patient and the greater or lesser amount of color indicates the greater or lesser number of patients in each neuron. c) Map components corresponding to the 16 variables considered: distribution of the values of study variables in each of the neurons that contain a pattern of patients with a similar minimum distance according to the Artificial Neural Network algorithm for the 16 variables considered. The variable analyzed appears over each map.

On analyzing the maps, it can be observed that several variables present the same information, for example, Agenesis 12 and Agenesis 22 and others with a practically symmetrical representation such as the lateral microdontia variable. Thus, these three variables were deleted and substituted by “development” (tooth-length) that had the value of 0 in the case of agenesis, 1 in the case of lateral microdontia and 2 if the tooth was complete. Other variables of similar behavior were initial ortho age and final ortho age, age_on and age_out. Moreover, the treatment type variable was also eliminated from the study as no correlation between neurons with greater prognosis of canine eruption with the treatment type was observed and, given that all were interceptive treatments, this was considered to be a variable that affected all the cases considered equally. Therefore, from this point on, the duration times of each individual treatment were not considered either. These variables remained represented by the initial age of the patient at the moment of beginning the treatment and treatment duration in which the time taken for all treatments employed is included. Following these considerations, we proceeded to undertake a second analysis in which the number of variables was now reduced to 16 (Fig. 3) : SEX, INITIAL AGE, SECTOR 13/23, LID, POSITION 13/23, TREATMENT DURATION, X21.21 FAN, EXFOL C+/+C, UNI/BI, NOLLA, INC 13/23, already shown in  Table 1 and adding the TOOTH LENGTH variable defined at the beginning of this paragraph.

On modifying the vector that characterizes each patient, the patterns are also modified and in this case the patients were distributed as shown in figure 3a, as well as cases of patients to whom fenestration was applied and the cases of normal eruption with orthodontic treatment (Fig. 3b). Finally, (Fig. 3c) shows, the 16 variables included in the study.

This graphic representation method allows us to follow the pattern characteristics of the patients that occupy each neuron. Neurons can be observed where the pattern corresponds to a high probability of eruption of the canine with no need for fenestration, or neurons where the pattern corresponds to a high probability of having to undertake a fenestration or neurons with an intermediate probability.

Thus, for example, in figure 3b, neuron 1, where 8 of the 9 patients included (88.9%) did not need fenestration for the eruption of the canines, presents a pattern similar to that shown in  Table 2. This table also shows the pattern of neuron 6 where 4 of the 6 patients included (66.6%) did need fenestration for the canines to erupt. These two neurons were selected for study, as they are consecutive and the difference of pattern between them can be clearly observed.

**Table 2:**
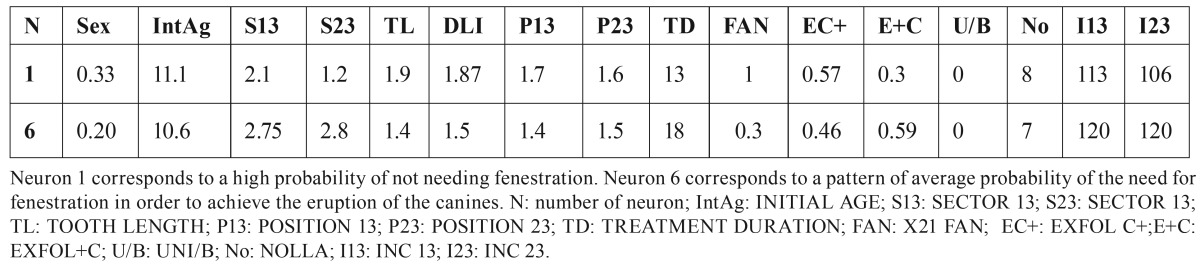
Average values of the patient pattern corresponding to each neuron (N) considered.

 Table 2. Average values of the patient pattern corresponding to each neuron (N) considered.

It can be observed that the value of several variables significantly differs between the two neurons analyzed, whereas in others the values are similar. To discover the pattern of patients according to the success of interceptive treatment for the eruption of the canine, we grouped the neurons together to form 3 clusters: Group 1 (GR1): made up of neurons 1, 7, 8, 10, 11, 14, 16, 22, 23 and 24 that represent a percentage of successful interceptive treatment of above 85%; Group 2 (GR2) is made up of neurons 4, 6, 9, 12, 13, 15, 16, 19, 20 and 25 in which the percentage of success was between 50 and 85% and Group 3 (GR3) made up of neurons 3, 5 and 21 where that percentage was below 50%.

 Table 3 shows the pattern corresponding to each of the groups considered and a variance analysis shows the variables that were significant when it came to distinguishing between these 3 groups of patients. The significance values for each variable are shown in  Table 3. In cases with a value of .000, these must be considered as *p*<0.001.

**Table 3:**
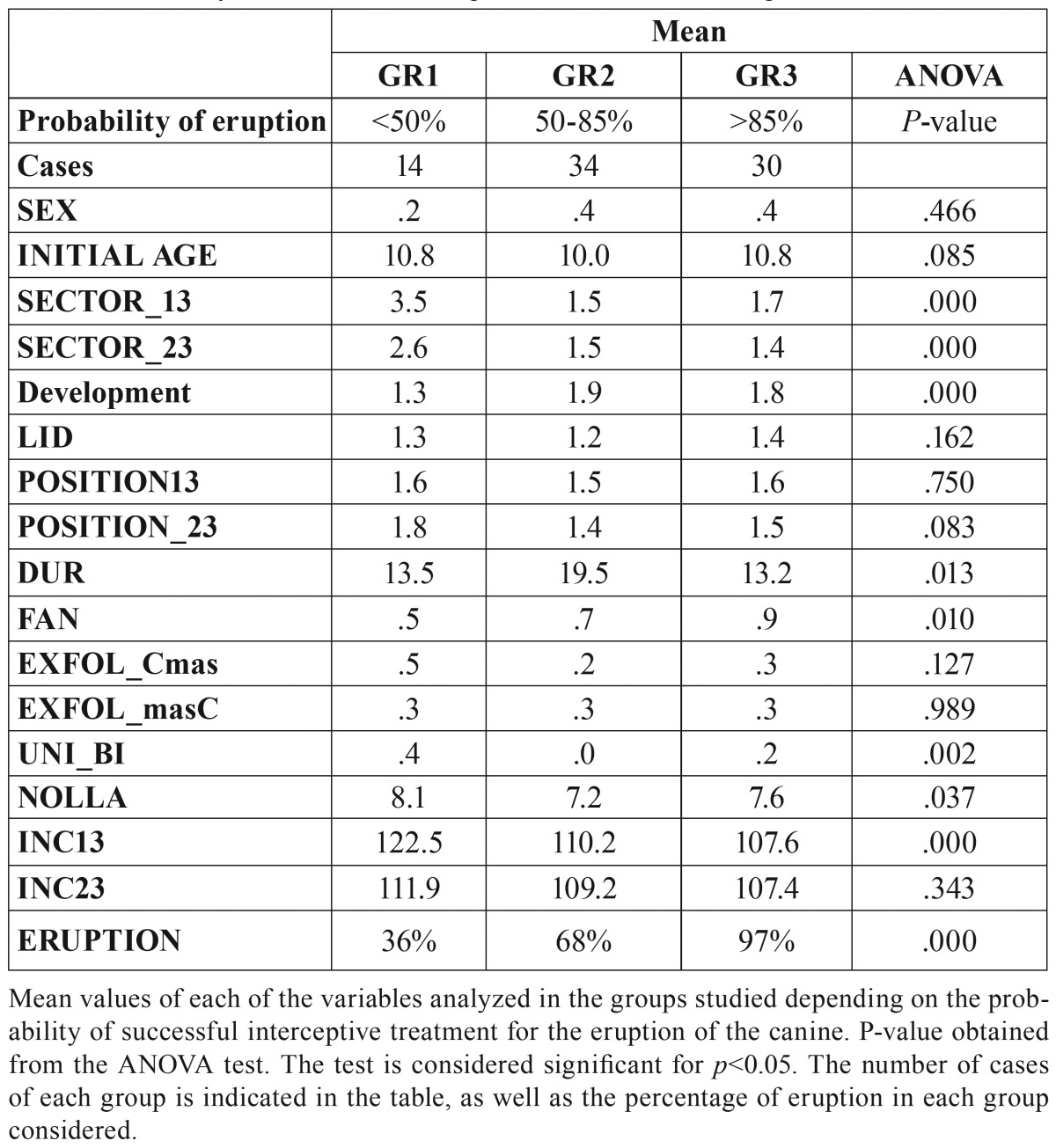
Probability of successful interceptive treatment for the eruption of the canine.

In our sample, GR1 group presented a probability of eruption of 36%, GR2 group 68% and the GR3 97% with a statistically significant difference between these percentages, these groups of patients, therefore, being suitable for characterizing the eruption pattern (<50%, between 50-85%, greater than 85%). On analyzing the pattern of each of these groups, it can be observed that the initial inclination of the canine and the sector where they are located are important prognosis factors as, at a greater inclination and greater sector, a greater probability of the need for fenestration arises. In fact, these results are also clear if a simple comparison of the mean values of patients who needed fenestration with those who did not is made. However, the methodology that we have employed allows us to add to these two factors the valuation of the rest of the variables that make up the pattern, some of which present statistically significant differences in terms of the probability of needing fenestration. The dependence of these other magnitudes does not appear when a customary statistics method comparing the means between the 2 groups is undertaken.

## Discussion

Although it is impossible to predict with absolute certainty which patients are going to require fenestration in order to achieve canine eruption, using the approach taken in this work, we present patterns of patients depending on their probability of either needing fenestration or not in order to achieve the eruption of the canine following an interceptive treatment.

We have found a pattern of low probability of achieving canine eruption employing only interceptive treatment (1st column of the table corresponding to < 50%) with the following values.

a) Sector values greater than 2.5.

b) Scant development of the lateral incisor.

c) Nolla stage greater than 8.

d) Inclination of the maxillary canine greater than 112º.

In our sample only 36-% of the patients with this pattern were successful in achieving canine eruption through interceptive treatment alone.

On the contrary, we found a pattern of high probability (>85 %) of not requiring a final fenestration to finish off the treatment. This pattern corresponds to the following values.

a) Sector values of 13 and 23 lower than 1.7.

b) Highly developed lateral incisor.

c) Nolla stage lower than 7.6.

d) Inclination of the lower canine less than 107º.

Although most studies focus on the possibility of the impaction or not of the PMC, and to explain this alteration numerous variables have been presented, not all are significant when it comes to explaining the need for a final surgical solution to carry the PMC to its correct final location. Hence, for example, the PMC alteration that presents itself most often in girls does not mean that this group is going to require a greater percentage of fenestrations. Likewise, other variables of interest in studies on impacted canines, such as age at beginning of treatment, coronal development of the lateral incisor; LID; the palatal or vestibular position of the canine and exfoliation of the deciduous canine that some consider effective (8), are of no importance to our study when it comes to predicting whether fenestration is going to be required or not. Even the duration of the treatment, which does show a statistically significant difference between the groups, as shown in table 3, is not representative for our study, as no difference exists in the cases of high and low probability.

However, the “sector, inclination, fan, Nolla stage” sectors can indeed predict the need or not for fenestration. These are also the variables that show a greater relationship with canine impaction in the different studies reviewed. Thus, in 1992 Lindauer *et al.* (3) used the same sectors as in the article of Wardorf *et al.* (13) for early identification of canine impaction. According to these authors, 78% of impacted canines were in sectors II, III and IV, i.e. that the crown of the canine was, at least, in contact or crossing the longitudinal axis of the root of the adjacent lateral incisor. Wardorf *et al.* (13)concluded that the “sector” variable is the best method for predicting canine impaction in the palate and that angulation did not increase the prediction of impaction. In this study, 82% of impactions were located in sectors II, III and IV. In our study we were able to confirm that, in the pattern with the greatest probability of the need for fenestration, this variable presents a mean value of 3, on considering the right and left canine, as against a value of 1.5 for the cases of low probability. It should be pointed out at this point that although we present mean values of the contralateral teeth, the right and left parts do not behave equally in our study: the right PMC is more frequently displaced and with greater intensity than the left PMC.

Wardorf *et al.* (13) also established that the mean angulation for impacted canines was 63.2, which corresponds to our measured angle of 116.8º. In our study, we were able to observe that the pattern of greatest probability of the need for fenestration corresponds to high levels of this angle, 117.2º on average as against 107.5º for the low probability pattern.

The development of the lateral incisor and Nolla stage also presented interesting results in our study, these variables being referred to in many canine impaction studies (1). In our case, the need for fenestration is associated with little-developed lateral incisors and lower Nolla stages. Becker and Chaushu (21) also sustain this eruptive theory in their article of 2015, as they observed that half the patients with impacted canines in the palate presented late development of dentition (a mean of 1.5 years), which is associated with Nolla stages lower than 7.6 and little-developed.

One variable, which nevertheless, has had little repercussion on the studies of impacted canines, “fanning” (of the lateral incisor) was representative in our study (2).

Broadbent as long ago as 1941, described the mechanism of eruption and alignment of the maxillary front teeth (22). This temporary arrangement of the fanned incisors he qualified as the “ugly duckling” stage through which the incisors acquire their fanned out position (apices together and, therefore, gapped or separated) crowns until they correct themselves as the canine eruptions take place.

Apart from evaluating the dental variables analyzed, we believe that the interesting point about this study resides in the fact that it shows the advantages gained by using new methodologies for representing complex clinical problems involving a large number of variables. Sets containing a large number of variables provide a better way of finding solutions to those problems. This methodology has allowed us to group together eruptive behavior patterns and to observe the pattern of patients by using a set of many of the variables involved, as well as to eliminate various variables that are redundant to the problem or of very scant repercussion.

## Conclusions

Unlike conventional statistics, self-organizing maps allow us to observe the problem of canine impaction and the need or not for fenestration, showing us how the variables are grouped together in one or the other of the different suppositions. We can assess the repercussion of each variable without the need to attend to concrete and absolute data by determining which set of variables appears to be associated with the greatest proportion for each clinical situation in our case.

Hence, we can predict more faithfully the different types of treatment prognosis, and, therefore, arrive at a safer diagnosis for future similar situations.
